# Relationship between Neural Crest Cells and Cranial Mesoderm during Head Muscle Development

**DOI:** 10.1371/journal.pone.0004381

**Published:** 2009-02-09

**Authors:** Julien Grenier, Marie-Aimée Teillet, Raphaëlle Grifone, Robert G. Kelly, Delphine Duprez

**Affiliations:** 1 CNRS, UMR 7622 Biologie Moléculaire et Cellulaire du Développement, Université Pierre et Marie Curie, Paris, France; 2 Developmental Biology Institute of Marseilles-Luminy, UMR CNRS 6216 Université de la Méditeranée, Marseille, France; Katholieke Universiteit Leuven, Belgium

## Abstract

**Background:**

In vertebrates, the skeletal elements of the jaw, together with the connective tissues and tendons, originate from neural crest cells, while the associated muscles derive mainly from cranial mesoderm. Previous studies have shown that neural crest cells migrate in close association with cranial mesoderm and then circumscribe but do not penetrate the core of muscle precursor cells of the branchial arches at early stages of development, thus defining a sharp boundary between neural crest cells and mesodermal muscle progenitor cells. Tendons constitute one of the neural crest derivatives likely to interact with muscle formation. However, head tendon formation has not been studied, nor have tendon and muscle interactions in the head.

**Methodology/Principal Findings:**

Reinvestigation of the relationship between cranial neural crest cells and muscle precursor cells during development of the first branchial arch, using quail/chick chimeras and molecular markers revealed several novel features concerning the interface between neural crest cells and mesoderm. We observed that neural crest cells migrate into the cephalic mesoderm containing myogenic precursor cells, leading to the presence of neural crest cells inside the mesodermal core of the first branchial arch. We have also established that all the forming tendons associated with branchiomeric and eye muscles are of neural crest origin and express the *Scleraxis* marker in chick and mouse embryos. Moreover, analysis of *Scleraxis* expression in the absence of branchiomeric muscles in *Tbx1^−/−^* mutant mice, showed that muscles are not necessary for the initiation of tendon formation but are required for further tendon development.

**Conclusions/Significance:**

This results show that neural crest cells and muscle progenitor cells are more extensively mixed than previously believed during arch development. In addition, our results show that interactions between muscles and tendons during craniofacial development are similar to those observed in the limb, despite the distinct embryological origin of these cell types in the head.

## Introduction

Craniofacial development requires the orchestrated integration of multiple tissue interactions. Defining the spatial relationship and the interactions between neural crest cells and muscle cells and their derivatives during jaw development is an important step towards understanding craniofacial malformations.

Jaws originate from the bilateral first branchial arches. The first arch gives rise to the maxillary and mandibular prominences and subsequently to musculo-skeletal structures of the upper and lower jaws [1], [2]. More caudally, the other branchial arches will provide the neck and throat components. Branchial arches are composed of pharyngeal endoderm, surface ectoderm, and two mesenchymal cell populations, originating from the neural crest and from cranial mesoderm, respectively. The ectodermal and endodermal components envelope the two mesenchymal cell types. Mapping of the cephalic neural folds, using quail chick chimeras, retroviral and DiI injections have shown that neural crest cells filling the branchial arches give rise to all the skeletal elements, connective tissues and tendons of the jaw, while the mesodermal core gives rise to myogenic cells of the jaw muscles [3]–[10].

Although previous fate mapping experiments have identified the majority of derivatives of neural crest cells and cranial mesoderm in the jaw, the spatial relationships and the interactions over time between both cell types are not completely understood. Neural crest cells colonising the first branchial arches originate from the posterior mesencephalon to rhombomere 3 [8], [11], [12]. Neural crest cells have been described as migrating in between the overlying surface ectoderm and the cephalic mesoderm (containing the myogenic progenitors), effectively separating these two tissues. Then, cephalic mesodermal cells and neural crest cells expand ventrally at the same time into the future branchial arch region. It has been described that neural crest cells envelop but initially do not penetrate the centrally located muscle plate of the branchial arches. Subsequently, coincident with muscle segregation, each muscle plate becomes infiltrated by neural crest cells, which may provide the muscle connective tissue of muscles, reviewed in [10], [13], [14]. Consequently, throughout their migration and subsequent organisation, neural crest cells are in close contact with the myogenic precursor cells during arch development. These extended interfaces between both cell populations have being suspected to be important for cell interactions during arch development and subsequent jaw morphogenesis.

Muscle formation relies on intrinsic program and extrinsic cues. The genetic program controlling head muscle specification is distinct from that underlying trunk and limb myogenesis, reviewed in [15]. Moreover, specific genetic programs drive the specification of head muscles, highlighting a genetic heterogeneity underlying head muscle development. *MyoR* and *Capsulin* regulate an initial step in the specification of a specific subset of branchiomeric muscles, the major muscles of mastication, derived from the first branchial arch [16]. The absence of *Tbx1* function in *Tbx1* ^−/−^ mutant mice, severely perturbs branchiomeric muscle development, while extra-ocular and body muscles are unaffected [17], [18]. *Pitx2* is expressed before *MyoR*, *Capsulin* and *Tbx1* and is required for their expression in the first branchial arch, although a second study has revealed *Pitx2* independent *Tbx1* expression [19], [20]. Although there is an emerging network of transcription factors involved in branchial arch muscle formation, we still do not have a global picture of the intrinsic developmental program responsible for the specification of different head muscles. In addition to the intrinsic genetic program, classical experiments in avian embryos have shown that head muscle formation is also dependent on extrinsic tissues [21], [22]. It has long been suspected that cranial neural crest cells influence head muscle formation [5], [23], [24]. However, the early steps of facial muscle specification have been shown to be independent of the presence of neural crest cells, since ablation of cranial neural crest cells in chick and amphibian embryos does not block initiation of the myogenic program in the branchial arches [21], [25]–[27]. All muscle genes reflecting early steps of the myogenic program, including *Capsulin*, *Tbx1*, *MyoR*, *Myf5*, *MyoD* and *desmin* are expressed in the branchial arches following neural crest cell ablation in chick embryos, although their expression domains are altered [21], [27]. The muscle differentiation program based on myosin expression is also initiated in crest-ablated arches; indicating that neural crest cells are not necessary to initiate muscle differentiation [21], [27]. However, in the absence of neural crest cells, jaw muscles were found to be severely reduced showing the requirement of neural crest cells for normal muscle organisation after the onset of muscle specification and differentiation [26], [27]. These experiments indicate an early neural crest cell-independent phase and then a later -dependent phase for branchiomeric muscle formation.

Branchiomeric muscle formation has been studied in the absence of neural crest cells [21], [27], but we do not know how neural crest cells behave in the absence of muscle. Tendons are one of the neural crest cell-derived tissues likely to interact with forming jaw muscles. Tendons did not attract much attention from developmental biologists probably due to the lack of early markers. The discovery that the gene encoding the bHLH transcription factor Scleraxis is expressed in embryonic tendons in body and limb muscles provided a major step forward [28]–[30]. Genetic ablation of *Scleraxis* in the mouse leads to defective differentiation of limb muscle tendons [31]. Although no head phenotype has been reported to date in the *Scleraxis* mutant mice [31], *Scleraxis* is expressed in the branchial arches and head tendons of mouse embryos [30], [32].

In the present study, we have reinvestigated the relationship between neural crest cells and myogenic cells (both mesodermal progenitors and differentiated muscle cells) during development of the first branchial arch. Using molecular and embryological markers, we observe an unexpected intermingling between myogenic cells and neural crest cells at various stages of development. Furthermore, we have show that all tendons in the embryonic head are of neural crest cell origin and express *Scleraxis*. By analysing tendon formation in absence of muscle using a genetic mouse model displaying loss of branchiomeric muscles, we were able to demonstrate that tendons initiate their development independently of muscles but that muscle is required for further tendon development.

## Materials and Methods

### Chick and quail embryos

Animals were treated according to French institutional guidelines. Fertilized chick eggs from commercial source JA 57 strain [Institut de Sélection Animale (ISA), Lyon, France] and Japanese quail eggs (Chanteloup, France) were incubated at 38°C. Before E2, embryos were staged according to somite number. Older embryos (E3–E7) were staged according to Hamburger and Hamilton (HH) [33]. The following day numbers and HH stages are equivalent: HH13/E2, HH20/E3, HH21/E3.5, HH22/E4, HH24/E4.5, HH26/E5, HH27/E5.5, HH28/E6, HH30/E7.

### Quail into chick grafting experiments

Quail and chick embryos were allowed to grow until 5–6 somite stages, when cephalic neural folds have formed. At this stage, neural crest cells have not yet migrated [34]. Chick neural lips from the first somite level to the diencephalic regions were excised and replaced by their quail counterparts [35]. Quail-chick chimeras were allowed to grow for another eight hours to five days more and treated for tissue sections and in situ hybridization.

### Mouse strains

Animals were treated according to French institutional guidelines. Mice carrying the *Tbx1* null allele (here referred to as *Tbx1* ^−/−^) were kindly provided by Virginia Papaioannou (Columbia University, New York) and mice and embryos were genotyped as described in [36]. Mice carrying the *yMyf5-nLacZ-96-16* transgene expressing the nlacZ reporter gene under the control of *Myf5* regulatory sequences have been described in [37]. Embryos were dated taking the day of the vaginal plug as E0.5.

### In situ hybridization to wholemount and to tissue sections

Normal or experimental embryos were fixed in Farnoy (30% (V/V) Formaldehyde 37,5%, 60% (V/V) Ethanol, 10% (V/V) acetic acid) and processed for in situ hybridization to 8 µm wax tissue sections as previously described [38]. Alternating serial sections from embryos were hybridized with probe 1 and probe 2. Pair of sections in the results comparing two probes is always strictly adjacent. For wholemount in situ hybridization, embryos were fixed with 4% (V/V) formaldehyde and processed as previously described [38]. The digoxigenin-labelled mRNA probes were used as described: chick and mouse *MyoD* [39], chick *MyoR* [40], mouse *MyoR* [17], chick and mouse *Scleraxis* [39]. The probe for chick *AP2α* originates from the UMIST EST library [41].

### Immunochemistry

Chick differentiated muscle cells were detected on sections after in situ hybridization using the monoclonal antibody MF20 (Developmental Studies Hybridoma Bank). Quail cells were detected using the QCPN antibody (Developmental Studies Hybridoma Bank) either directly on 8 µm wax sections or after in situ hybridization. Nerve cells were detected using the HNK1 antibody [42]. Immunochemistry on cryostat sections through mouse embryos were performed as described in [32]. Specific antibodies against β-galactosidase (polyclonal, 1/2000, Capel) and AP2α (polyclonal, 1/25, Developmental Studies Hybridoma Bank) were used to detect β-galactosidase and mouse AP2α proteins, respectively, in branchial arches of *yMyf5-nLacZ-96-16* transgenic embryos.

## Results

### *MyoR* and *MyoD* expression in head muscles

**Figure 1:**
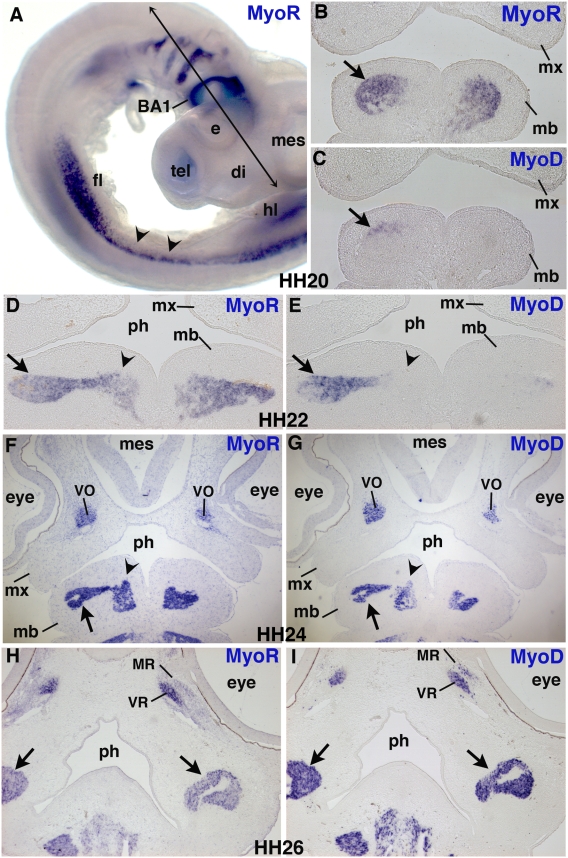
Comparison of *MyoR* and *MyoD* expression domains during chick first branchial arch development. (A) Lateral view of a HH20 chick embryo hybridized with a *MyoR* probe. HH20 (B,C), HH22 (D,E) HH24 (F,G) and HH26 (H,I) embryos were frontally sectioned at the head level. The plane of section is indicated in the panel A. Adjacent sections from each stage were hybridized with DIG-labelled antisense probes for either *MyoR* (B,D,F,H) or *MyoD* (C,E,G,I). *MyoR* delineates the myogenic core of the first branchial arch (B,D,F) and is subsequently expressed in all branchiomeric muscles (H). *MyoD* transcripts are first detected in a lateral sub-region of the *MyoR* domain at HH20 (B,C, arrows) and HH22 (D,E, arrows), then spread progressively from lateral to medial regions to overlap with the *MyoR* domain in branchiomeric muscles (E,G,I). (D–G) Arrows point to the lateral domains of the core, while arrowheads show the medial domain of the core. (H,I) Arrows indicate the branchiomeric muscles expressing both *MyoR* (H) and *MyoD* (I). (A) Arrowheads indicate the hypaxial lips at the interlimb level, expressing *MyoR*. BA1, first branchial arch, di, diencephalon; e, eye; fl, forelimb; hl, hindlimb; mb, mandibular arch; mes, mesenchephalon; MR, medial rectus; mx, maxillary arch; ph, pharynx; tel, telencephalon; VO, ventral oblique; VR, ventral rectus.

In order to analyse the neural crest cell/muscle interface during branchial arch development, we needed to define an early marker for muscle progenitors. We chose *MyoR*, since it has been described as being expressed in developing branchiomeric muscles in chick embryos [40]. In order to precisely define the *MyoR* expression domain in relation to the various steps of muscle formation, we compared its expression with that of *MyoD* by in situ hybridization to adjacent tissue sections, during development of the first branchial arch. At HH20, *MyoR* was strongly expressed in the chick branchial arches in addition to the hypaxial lips of the interlimb somites and muscle progenitors migrating to the limb buds (Figure. 1A), [40]. The first branchial arch undergoes profound rearrangements leading to formation of the upper maxillary and the lower mandibular prominences. At HH20, *MyoR* transcripts were detected in a large part of the mesenchyme constituting the so-called myogenic core of the branchial arch (Figure 1B). *MyoD* expression was first detected at this stage in a sub-domain of the *MyoR*-positive region, corresponding to the upper and lateral part of the mandibular process of the first branchial arch (arrows in Figure 1B,C). At HH22, the *MyoR* expression domain is still larger than that of *MyoD* in the mandibular process of the first branchial arch (Figure 1D,E, arrows and arrowheads). At HH24, the muscle plate strongly expressed *MyoD* in the lateral part of the mandibular buds; *MyoR* expression was maintained in this region (Figure 1F,G, arrows). In addition, *MyoD* began to be expressed in the medial area of the branchial arch, in a more discrete domain than that of *MyoR* (Figure 1F,G, arrowheads). At HH26, the head of the avian embryo has grown considerably and the muscle plates have been profoundly rearranged, leading to the individualization of branchiomeric-derived muscles, the jaw operating muscles. At this stage, *MyoR* and *MyoD* expression domains overlapped completely in all branchiomeric muscles (Figure 1H,I, arrows). In summary, *MyoR* is expressed before *MyoD* in the myogenic core of the branchial arches. From HH20, the *MyoD* expression domain progressively spreads out to overlap with that of *MyoR*, which provides a good marker for myogenic precursor cells in branchial arches.

**Figure 2:**
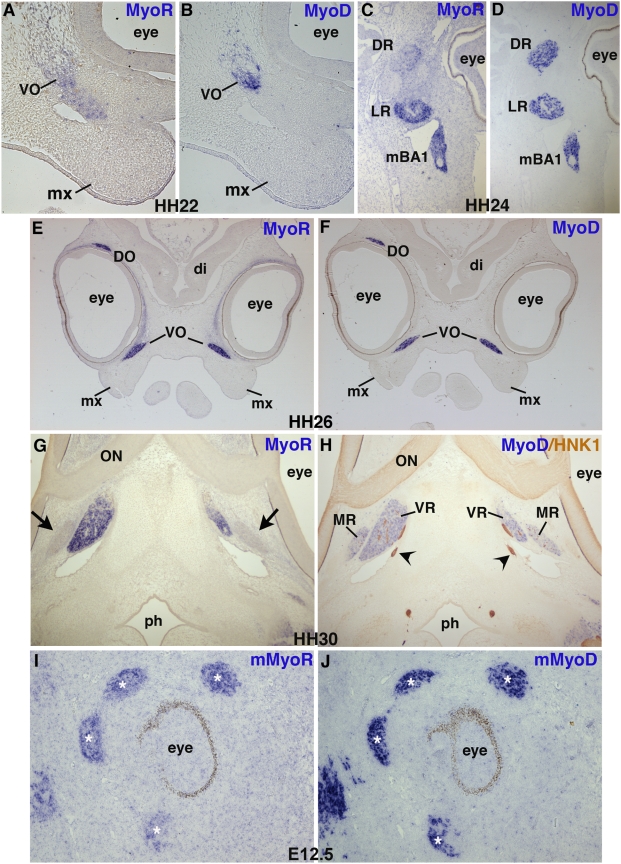
Comparison of *MyoR* and *MyoD* expression in extra-ocular muscles. HH22 (A,B), HH24 (C,D), HH26 (E,F), HH30 (G,H) chick embryos were frontally sectioned at the head level. Adjacent sections from each stage were hybridized with DIG-labelled antisense probes for either *MyoR* (A,C,E,G) or *MyoD* (B,D,F,H). E12.5 mouse embryos were sagitally sectioned at the head level and hybridized with DIG-labelled antisense probes for either *mMyoR* (I) or *mMyoD* (J). In the chick embryo, At HH22, the ventral oblique is the first ocular muscle to express *MyoR* and *MyoD* (A,B). At HH 24, *MyoR* transcripts are observed faintly in the dorsal rectus, strongly in lateral rectus (C), while *MyoD* is expressed in both dorsal rectus and lateral rectus muscles and in branchiomeric muscles (D). At HH26 stage, the ventral and dorsal obliques harbour strong *MyoR* (E) and *MyoD* (F) expression. At HH30, when the medial and ventral rectus muscles are individualized, *MyoD* is expressed in both muscles, while *MyoR* is expressed in ventral rectus but not in medial rectus (G,H). (H) The innervation is labelled with HNK1 antibody, two arrowheads point to nerves. The optic nerve is also labelled in light brown with the HNK1 antibody. (G) The absence of *MyoR* expression in the medial rectus is indicated by arrows. (I,J) In E12.5 mouse embryos, extra ocular muscles, labelled by white asterisks (J) expressed both *mMyoR* (I) and *mMyoD* (J). di, diencephalon; DO, dorsal oblique; DR, dorsal rectus; LR, lateral rectus; mBA1, first branchial arch muscles; MR, medial rectus; mx, maxillary arch; ON, optic nerve; ph, pharynx; VO, ventral oblique; VR, ventral rectus.

We extended our analysis of facial muscle emergence by analysing *MyoR* and *MyoD* expression in extra-ocular muscles. Extra-ocular muscles comprise six muscles, namely ventral oblique, dorsal oblique, lateral rectus, dorsal rectus, medial rectus and ventral rectus. Despite their common final location site near the eyes, extra-ocular muscles derive from distinct parts of the mesoderm, the lateral rectus and dorsal oblique originate from cephalic mesoderm, while the other 4 extra-ocular muscles originate from prechordal head mesoderm [5], [10] and reviewed in [14]. In addition, the lateral rectus muscle has a distinct genetic program from that of the other extra-ocular muscles, since it is the only facial muscle, which expresses *Pax3* and *Lbx1* [43]. In the head, in contrast to the exclusive association described for *MyoR* and branchiomeric muscles [16], [40], we also observed *MyoR* expression in extra-ocular muscles (Figure 2). At stage HH22, *MyoR* was expressed in the ventral and dorsal oblique muscles, in a larger domain than that of *MyoD* (Figure 2A,B and data not shown, see also Figure 1F,G for HH24). However, at HH26 *MyoR* and *MyoD* expression domains overlapped in both ventral and dorsal obliques (Figure 2. E,F). In the dorsal rectus, *MyoD* expression was broader than *MyoR*, while the lateral rectus expresses both genes (Figure 2C,D). The medial rectus was the only extra-ocular muscle, which did not express *MyoR* (Figure 2G,H, see also Figure 1H,I). *MyoR* transcripts were also observed in extra-ocular muscles in mouse embryos at E11.5 and E12.5, in addition to being expressed in branchiomeric muscles (Figure 2I,J, asterisks and data not shown). However, *MyoR* expression was no longer observed in any mouse head muscles at E15.5 (data not shown). In summary, *MyoR* is not an exclusive marker of branchiomeric muscles as previously described [16], [40], but also labels extra ocular muscles in both chick and mouse embryos.

### The relationship between neural crest cells and myogenic mesodermal cells in the first branchial arch

**Figure 3:**
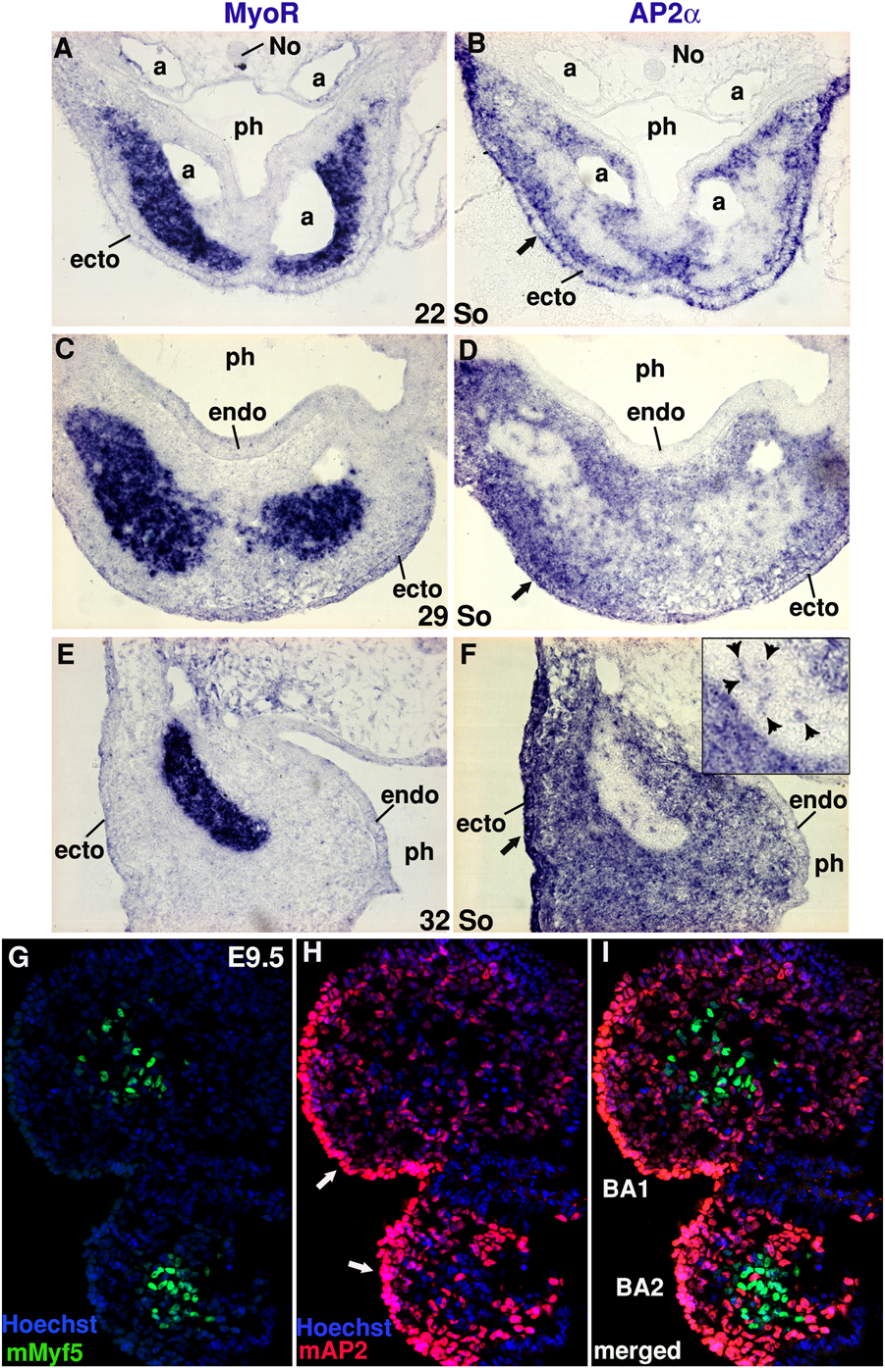
Neural crest cells visualized with *AP2α* expression are observed inside the mesodermal core of the first branchial arch. Adjacent transverse sections of chick embryos at the level of the first branchial arch at 22 (A,B), 29 (C,D) and 32 (E,F) somite-stages were hybridized with *MyoR* (A,C,E) and *AP2*α (B,D,F) probes. The mesodermal core is visualized with *MyoR* expression (A,C,E). *AP2α* is expressed in all neural crest cells within the arch and in the surface ectoderm (B,D,F). The *AP2α*-positive surface ectoderm is arrowed in (B,D,F). *AP2 α* positive cells are also observed inside the *MyoR*-positive domain (B,D,F). The inset in F shows an enlargement of the mesodermal core, where the *AP2 α-*positive cells are indicated by arrowheads. Frontal sections of E9.5 mouse embryos from *Myf5-nlacZ* mice were immunostained using an anti-β-galactosidase antibody to visualise *mMyf5* expression (G, green) and an anti-AP2 antibody to detect mAP2*α* location (H, red). I is a merged picture of G and H. AP*2α*-positive cells (red) are observed in the mesodermal core delineated by *Myf5*-postive cells (green). Hoechst staining in blue indicate nuclei. a, aortic arch; BA1, first branchial arch; BA2, second branchial arch; ecto, surface ectoderm; endo, pharyngeal endoderm; No, notochord; ph, pharynx.

The current model describing the spatial relationship between neural crest cells and myogenic precursor cells during branchial arch development assumes that neural crest cells first circumscribe, without penetrating, the condensed core of muscle precursors, reviewed in [14]. Using specific molecular markers for muscle precursors and neural crest cells, we did not observe such a scheme (Figure 3). We used *MyoR* to label myogenic progenitors of branchiomeric muscles (Figure 1), [40]. As a neural crest cell marker, we used *AP2α* (Activating Protein-2*α*), a transcription factor involved in face and limb morphogenesis [44]–[47]. These two markers permitted analysis of the respective location of muscle precursor cells and neural crest cells in the first branchial arch of chick embryos between 22 and 32 somites. At these developmental stages, *MyoR* expression defined the myogenic core (Figure 3A,C,E). As expected, *AP2*-positive cells surrounded the *MyoR*-positive domain and filled the entire space between the ectoderm and the endoderm. However, we also observed a significant number of *AP2*-positive cells inside the *MyoR-*positive mesodermal core (Figure 3B,D,F). *AP2* expression is also expressed in the covering ectoderm (Figure 3B,C,F, black arrows) as previously described [46], [48]. This result suggests that neural crest cells intermingle with myogenic precursor cells early during branchial arch development. Analysis of E9.5 mouse branchial arches identified *mAP2*-positive cells within the myogenic cores of the first and second branchial arches, labelled by expression of a *mMyf5* reporter transgene (Figure 3G–I). Early infiltration of the mesodermal core by neural crest cells therefore occurs in both species.

**Figure 4:**
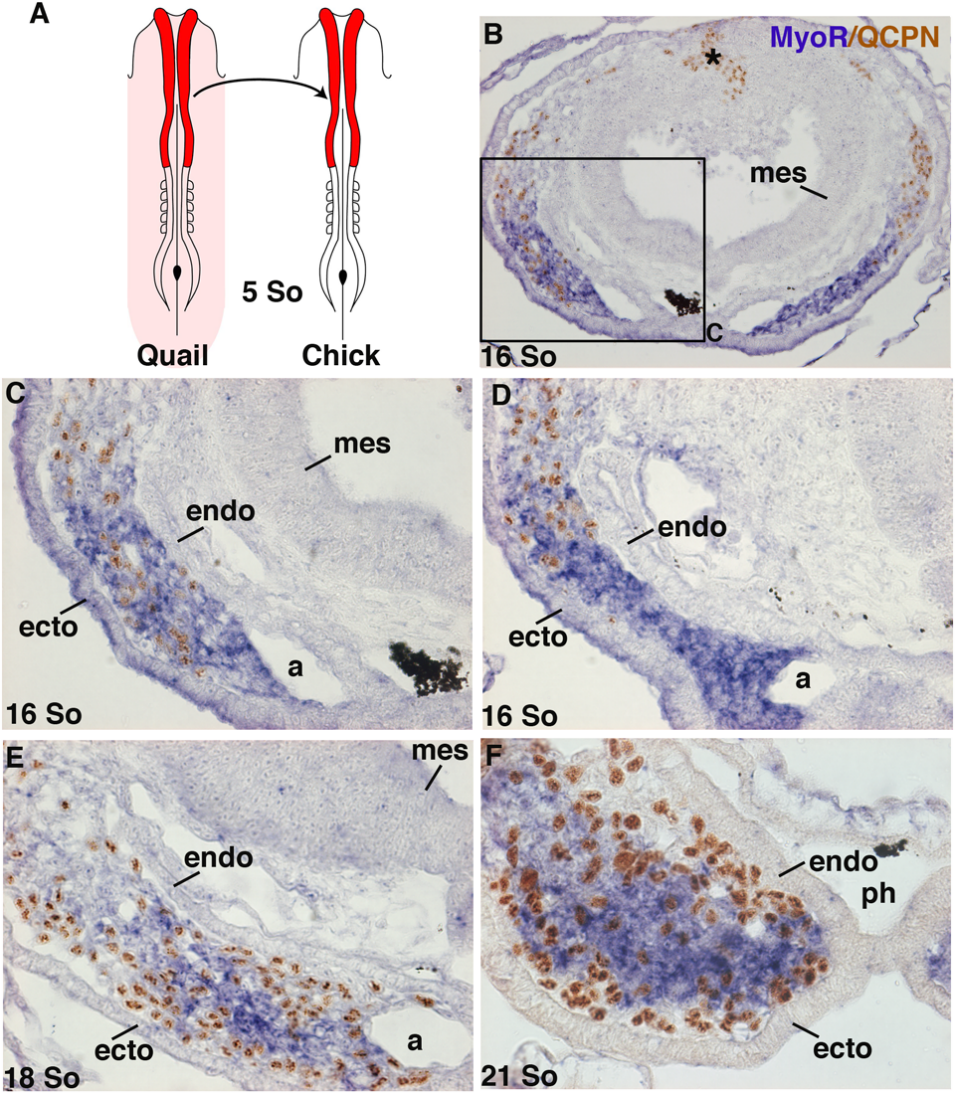
Neural crest cells invade the cephalic mesoderm. (A) Schematic representation of the chick neural fold replacement by its quail counterpart (red) performed on 5/6-somite stage quail and chick embryos. (B–F) Transverse sections of quail–chick chimeras at 16 (B,C,D), 18 (E) and 21 (F) somite-stages, at the level of the future first branchial arch were hybridized with the *MyoR* probe followed by immunohistochemistry using the QCPN mAb. (C) is a higher magnification of (B). (B,C,D) corresponds to sections from the same embryo, (B,C) being slightly (80 µm) more rostral than (D). The asterisk in B marks the position of the graft. (B–D) At the future first branchial arch level, the QCPN-positive cells progressively invaded the cephalic mesoderm expressing *MyoR,* in a rostral to caudal manner. At 18 and 21 somite-stages (E,F), QCPN positive cells are observed inside the *MyoR*-positive domains. a, aortic arch; ecto, ectoderm; endo, pharyngeal endoderm; mes, mesencephalon; ph, pharynx.

In order to follow the neural crest cells in the first branchial arch other than with molecular markers, we replaced the cephalic neural folds (diencephalon, mesencephalon, anterior rhombencephalon) from chick embryos at 5–6 somite-stages, just before neural tube closure, with the quail equivalent (Figure 4A). This experiment replaces chick neural crest cells by their quail counterparts. Using the QCPN antibody, which specifically recognizes quail cells, combined with in situ hybridization using *MyoR* probe, we were able to analyse the behaviour of neural crest cells and muscle precursor cells during formation of the first branchial arch. Studies of 2 day-old chimeras between 16 and 21 somite-stages (HH12–HH14) permitted quail neural crest cells to be followed en route to the future branchial arch. At16 somite-stage, QCPN-positive cells are clearly observed within the *MyoR*-positive domain at the future arch level (Figure 4B,C). At a slightly more caudal level (80 µm), QCPN-positive cells are about to invade the *MyoR*-positive cephalic mesoderm (Figure 4D), consistent with the anterior to posterior progression of neural crest cell migration [34]. Three hours later (18 somite-stage) neural crest cells have totally invaded the mesenchyme of the forming branchial arch (Figure 4E). From the 18 somite-stage onwards, neural crest cells are intermingled with *MyoR*-positive cephalic mesoderm regardless of the axial level. In 21 somite-stage embryos, neural crest cells and mesoderm were still mixed, but neural crest cells have begun to accumulate close to the pharyngeal endoderm and surface ectoderm (Figure 4F), generating a physical frontier between the mesodermal core and pharyngeal epithelia. From this stage (21 somites), the head of the embryos begins to rotate and branchial arches start to expand. These results show that neural crest cells do not circumscribe the *MyoR*-positive domain of cephalic mesoderm but rather penetrate into this domain and intermingle with it. However, as branchial arch formation proceeds there is an increase of neural crest cell number close to the endoderm and ectoderm surrounding the myogenic core.

**Figure 5:**
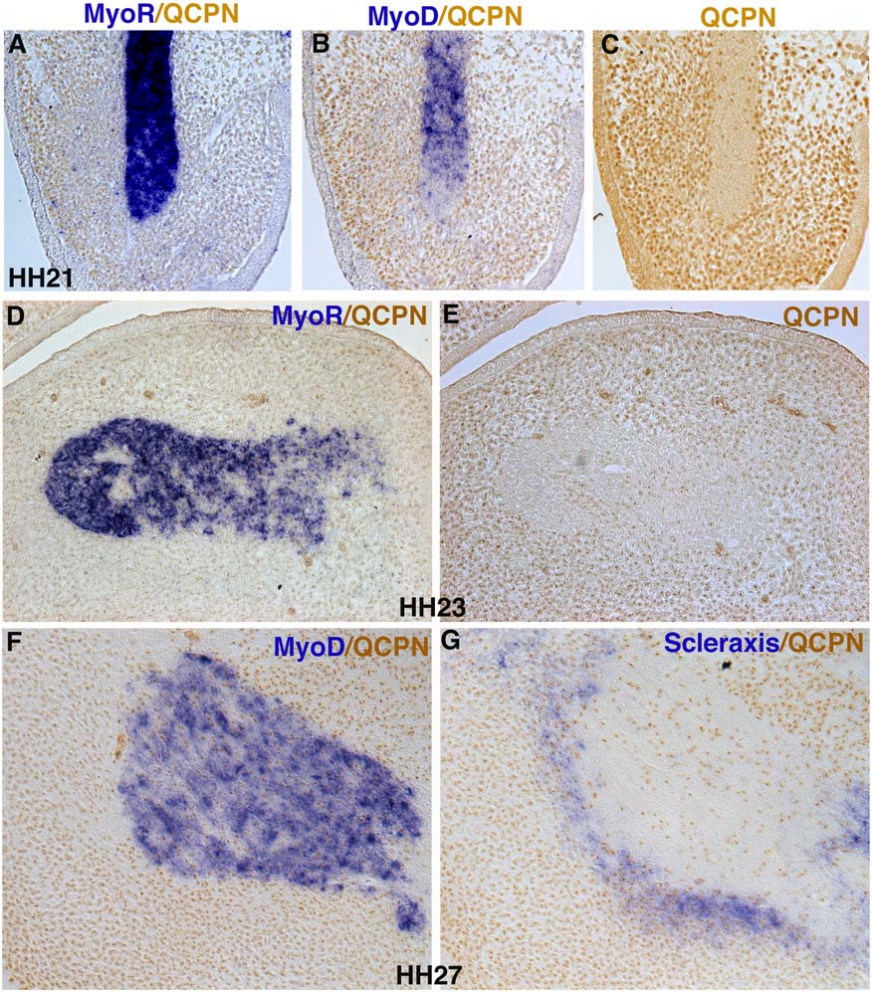
Relationship between neural crest cells and muscle precursor cells during branchial arch development. (A–C) Adjacent frontal sections from HH20 quail-chimeras at the first branchial level were hybridized with *MyoR* (A) and *MyoD* (B) probes and then incubated with QCPN antibody or uniquely incubated with QCPN antibody (C). (D–G) Adjacent frontal sections from HH23 (D,E) and HH27 (F,G) quail-chimeras at the first branchial level were hybridized with *MyoR* (D), *MyoD* (F) or *Scleraxis* (G) probes and then incubated with the QCPN antibody or directly incubated with the QCPN antibody (E). QCPN-positive cells are observed in the *MyoR*- and *MyoD*-positive domains of the core at HH21 (A–C) and HH23 (D,E). From HH27 there is an increase of QCPN-positive cells inside the muscles (F,G).

Twenty-four hours after grafting, the head of the embryo has grown considerably. As shown on frontal sections of HH21 chimeras (Figure 5A–C), neural crest cells were the major cell type present in first branchial arch mesenchyme. The myogenic plate was restricted to the central part of the arch, where *MyoD* expression began to be detectable in the *MyoR*-positive domain (Figure 5A,B). Nevertheless, neural crest cells continued to be observed in the muscle plate of the first branchial arch (Figure 5C). However, the density of QCPN-positive cells in the muscle plate is low compared to that in surrounding mesenchyme (Figure 5C), reflecting the massive expansion of neural crest cells outside the core. A similar situation is observed at HH23 (Figure 5D,E). After six days of development (HH27), profound rearrangements of the first branchial arch muscle plate have led to individualization of the jaw muscles. At this stage, there is a clear increase in neural crest cell density inside the muscle masses compared to previous stages (compare Figure 5G with Figure 5C,E). This expansion of QCPN-positive cells within branchiomeric muscles is concomitant with the appearance of *Scleraxis* expression in tendon primordia, which are of neural crest cell origin (Figure 5F,G). Neural crest cells inside the muscle masses may constitute the muscle connective tissue.

In summary, our molecular and cellular analyses during branchial arch development showed that neural crest cells first penetrate into the cephalic mesoderm, resulting in intermingling of neural crest and mesodermal cells at early stages. After two days of development, neural crest cells outside the forming mesodermal core expand dramatically leading to the outgrowth of the arch and to an obvious difference in neural crest cell density between the myogenic core and the rest of the branchial arch. It is only after muscle splitting has occurred, from HH27, that neural crest cells, likely providing the muscle connective tissue cells, expand within developing branchiomeric muscles.

### All forming head tendons of chick embryos express *Scleraxis*

**Figure 6:**
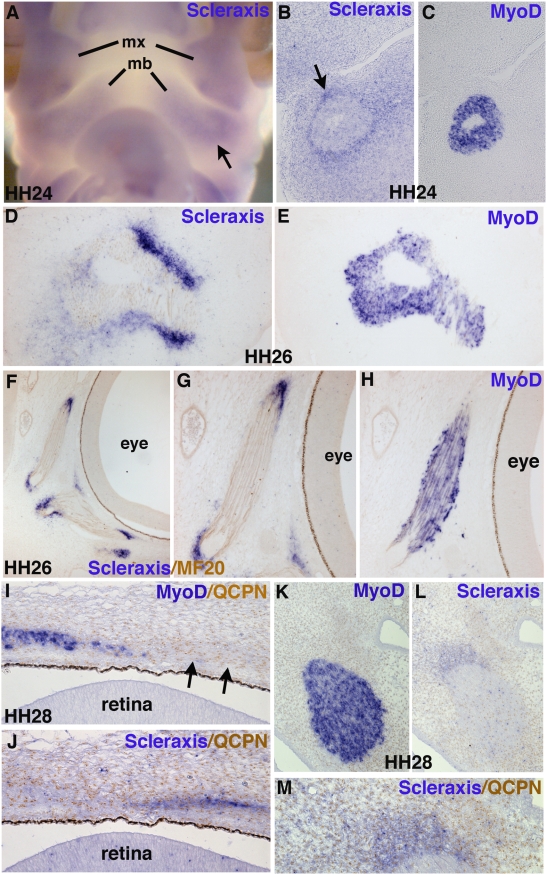
Head tendons express *Scleraxis* and are of quail origin. (A) In situ hybridization to HH24 embryos with the *Scleraxis* probe. Adjacent frontal sections of HH24 (B,C) and HH26 (D–H) embryos were hybridized with the *Scleraxis* (B,D,F,G) and *MyoD* (C,E,H) probes were followed by an immunohistochemistry using the MF20 antibody to reveal differentiated muscle fibres. MF20 is visible in (F,G). (A,B) Arrows point to the *Scleraxis* expression domain in tendon primordia at HH24. At HH26, *Scleraxis* labels branchiomeric tendons (D,E) and eye tendons (F–H). (I–M) Adjacent saggital sections of HH28 quail-chick chimeras, were hybridized with the *MyoD* (I,K) and *Scleraxis* (J,L,M) probes followed by immunohistochemistry using the QCPN antibody. QCPN is visible in (I,JM). The *Scleraxis*-positive tendons associated with the extra ocular muscle (I,J) or with a jaw operating–muscle (K–M) are of quail origin. (M) is a higher magnification of (L).

Tendon is a candidate tissue derived from neural crest cells susceptible to interact with muscle organisation. The best marker for tendon formation is the bHLH transcription factor, *Scleraxis* [28], [39]. Prior to HH24, expression of *Scleraxis* in head mesenchyme is general and diffuse, while *Scleraxis* expression clearly defines the syndetome domain within somites at the same stages (data not shown), [29]. At HH24, an increase of *Scleraxis* expression was observed in tendon primordia of the first branchial arches, surrounding the muscle masses labelled by *MyoD* (Figure 6A–C, arrows). The timing of *Scleraxis* expression in tendons associated with extra-ocular muscles is similar to that in branchiomeric tendons. By HH26, *Scleraxis* expression delineated forming tendons of all head muscles including the future jaw-operating muscles, the tongue and eye muscles (Figure 6D–H and data not shown). At HH30, *Scleraxis* expression is observed in all head tendons (data not shown). We conclude that *Scleraxis* labels all chick head tendons. However, its expression is detected later in head tendons compared to somite and limb tendons.

In order to demonstrate that the *Scleraxis* positive-tendons are of neural crest cell origin, we performed neural fold replacement in 5–6 somite-stage chick embryos by their quail counterparts as described previously (Figure 4A) and analysed *Scleraxis* expression in such chimeras. *Scleraxis* expression domains associated with extra-ocular muscles and branchiomeric muscles (Figure 6I–M, see also Figure 5G) are all constituted of quail cells, confirming that branchiomeric and eye tendons are of neural crest origin, as are the corresponding skeletal structures [34].

### Tendon and muscle interactions during branchial arch development

**Figure 7:**
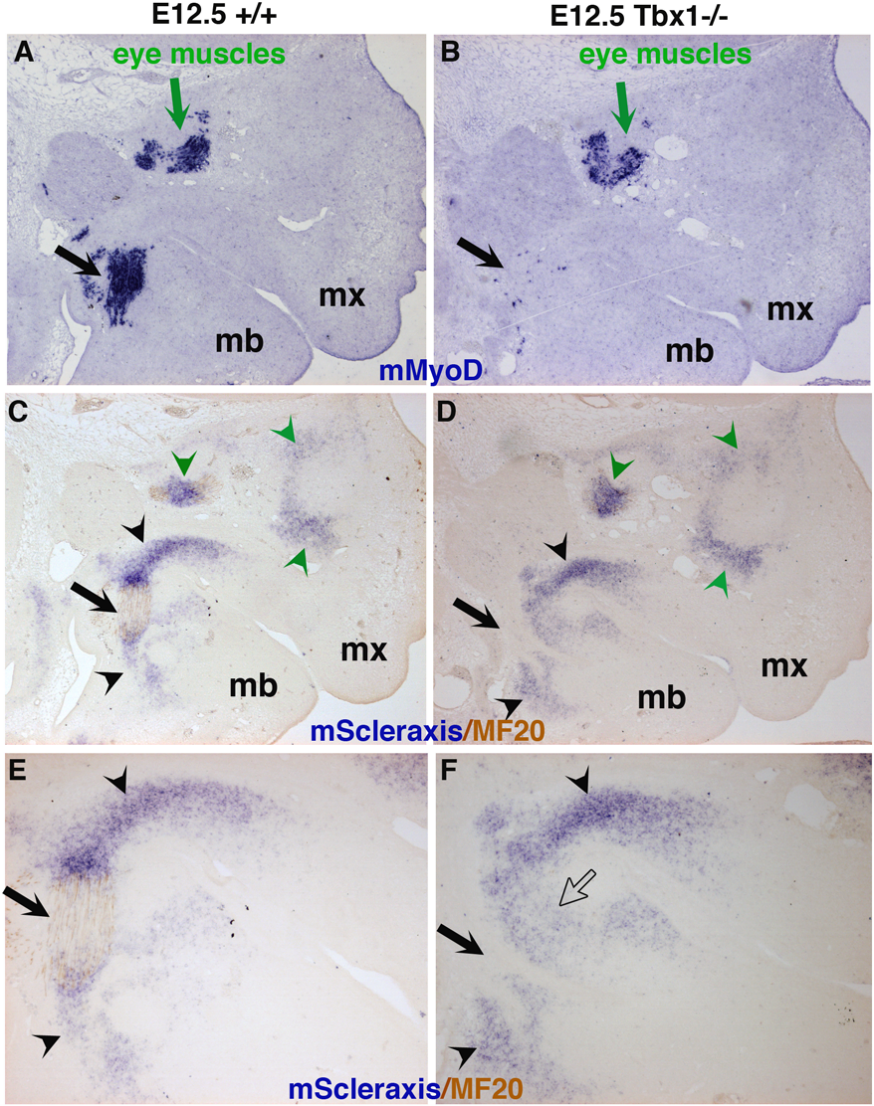
*Scleraxis* expression is normally established in the absence of differentiated muscles in the first branchial arch of E12.5 *Tbx1* ^−/−^ mice. Adjacent saggital sections of either wild-type (A,C,E) or *Tbx1* ^−/−^ mutant mice (B,D,F) were hybridized with *mMyoD* (A,B) or *mScleraxis* (C–F) probes. (C–F) After in situ hybridization with a mouse *Scleraxis* probe, the differentiated myofibres were detected using MF20 antibody. *Tbx1* ^−/−^ mutant mice display a loss of branchiomeric-derived muscles, highlighted by the absence of *mMyoD* expression (B, black arrow) and MF20 labelling (D, F black arrows) compared to the wild type situation (A,C,E, black arrows). Despite the absence of branchiomeric muscles, *mScleraxis* expression pattern remains unchanged in mutant mice (D,F black arrowheads) compared to wild-type (C,E black arrowheads). Green arrows (A,B) and arrowheads (C,D) point to the non-affected extraocular muscles and tendons, respectively in control (A,C) and *Tbx1* ^−/−^ mutant mice (B,D). (E,F) are high magnifications of (C,D) respectively. The open arrow in F indicates *Scleraxis* domain that has spread to the space left by the absent muscle.

**Figure 8:**
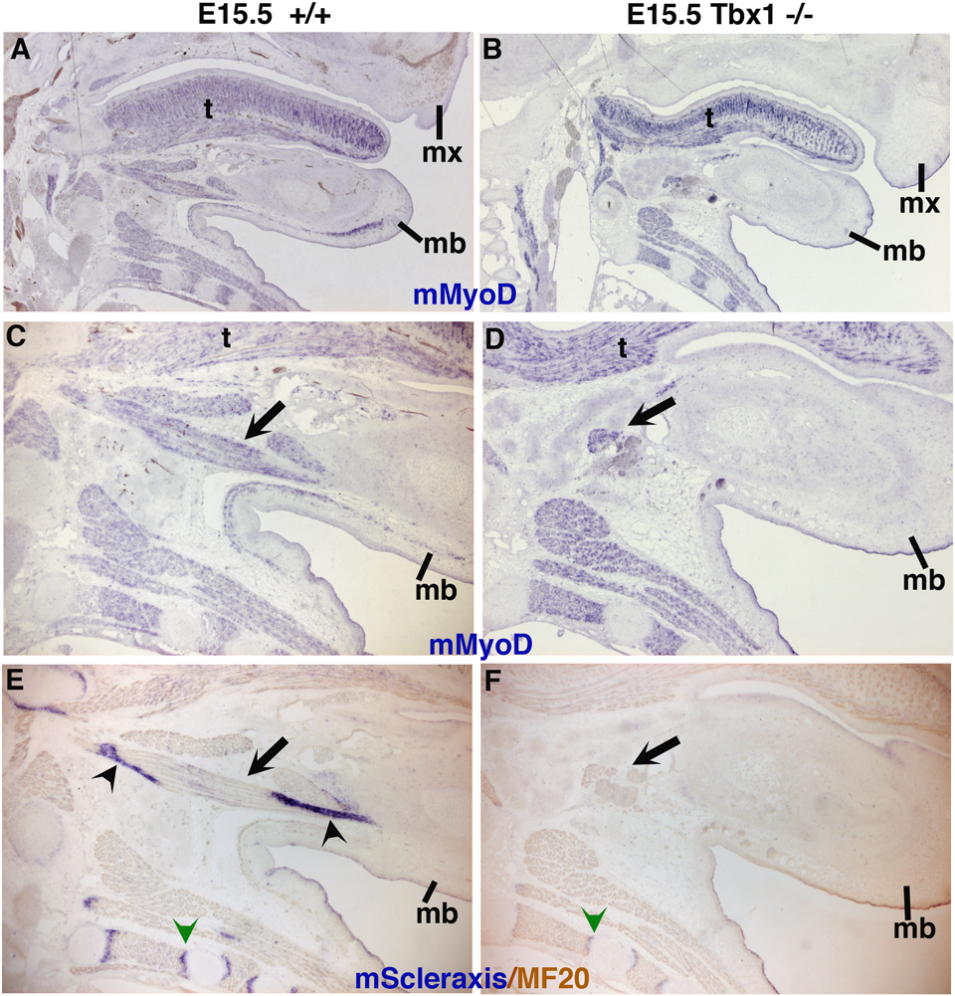
*Scleraxis* expression is lost in the absence of differentiated muscles in the first branchial arch of E15.5 *Tbx1* ^−/−^ mice. Adjacent saggital sections of either E15.5 wild-type (A,C,E) or E15.5 *Tbx1* ^−/−^ mutant mice (B,D,F) were hybridized with *mMyoD* (A–D) or *mScleraxis* (E,F) probes. (E,F) After in situ hybridization with a mouse *Scleraxis* probe (blue), differentiated myofibres were detected using MF20 antibody (light brown). (C,D) are higher magnification of A,B), respectively, focusing on the mandible. *Tbx1* ^−/−^ mutant mice display loss of branchiomeric muscles, highlighted by the absence of both *mMyoD* expression (B,D) and MF20 labelling (F) in the mandibula, compared to the wild type situation (A,C,E). The anterior digastric muscle is arrowed in the control mandible (C,E), while residual muscle masses are indicated by an arrow (D,F) in similar mandibular regions of *Tbx1^−/−^* mutant mice. Non-branchiomeric muscles are not affected in E15.5 *Tbx1* ^−/−^ embryos. (E) *Scleraxis* expression is observed in tendons associated with the anterior digastric muscle in the wild type situation (arrowheads). (F) *Scleraxis* expression is lost in the absence of muscles in E15.5 *Tbx1^−/−^* mutant mice, while *Scleraxis* expression is normally associated with non-branchiomeric muscles (green arrowheads). Mb, mandibular, mx, maxillary; t, tongue.

The study of chimeras revealed a strong physical intermingling between neural crest cells and mesodermal muscle cells in branchial arches. The influence of neural crest cells on branchiomeric muscle formation has been studied [21], [26], [27]. Conversely, we wanted to evaluate the relative importance of the presence of myogenic cells for tendon formation. In order to address this, we analysed *Scleraxis* expression in the absence of muscle cells. We used *Tbx1* ^−/−^ mice as a genetic model of loss of branchiomeric derived muscles [17]. In the absence of *Tbx1*, branchiomeric muscles fail to form, or are severely reduced in size, assayed by the quasi-absence of *MyoD* expression at sites of branchiomeric myogenesis in *Tbx1* ^−/−^ versus wild-type embryos at E12.5 (Figure 7A,B, black arrows), [17]. At this stage, *MyoR* expression is no longer detected at sites of branchiomeric muscle formation in *Tbx1* ^−/−^ embryos, whereas expression is observed normally in control embryos (data not shown). In contrast, extra-ocular muscles are unaffected despite the absence of *Tbx1* activity (Figure 7A,B, green arrows), [17]. In situ hybridization to tissue sections from mouse heads showed that *Scleraxis* transcripts were detected in all forming tendons of the head at E12.5 (Figure 7C,E and data not shown). In the first branchial arch, *Scleraxis* expression was restricted to the extremities of differentiated myofibres of branchiomeric muscles. In E12.5 *Tbx1* ^−/−^ embryos, the *Scleraxis* expression domain in the maxillary and mandibular prominences was indistinguishable from the normal situation (compare panels C,E with D,F respectively in Figure 7, tendons are indicated with black arrowheads), indicating that the initiation of *Scleraxis* expression was independent of branchiomeric muscle formation. However, at E15.5 *Scleraxis* expression was lost in the structures derived from the first branchial arch of *Tbx1* ^−/−^ embryos, in the absence of branchiomeric muscles (Figure 8). Arrowheads in Figure 8E point to the tendons associated with the anterior digastric muscle derived from the first branchial arch. In the *Tbx1^−/−^* mutant, this muscle is absent (compare Figure 8A,C with Figure 8B,D, respectively, arrows in C,D) and the associated tendons are not observed (Figure 8F). *Scleraxis* expression is however observed normally in tendons of extra-ocular and non-branchiomeric muscles of mutant embryos, in addition to regions where hypoplastic branchiomeric muscles were present (data not shown), [32]. These results showed that in the first branchial arch, muscles are not necessary for the initiation of one neural crest cell-derivative, tendons, but that muscles are required for further tendon development.

## Discussion

### Spatial relationships between neural crest cells, cranial mesoderm and their derivatives

The current consensus holds that neural crest cells do not mix with cranial mesoderm but rather migrate superficially to the mesoderm and underneath the overlying surface ectoderm and then envelop the mesoderm-derived myogenic cores in the branchial arches, thus leading to the formation of a sharp neural crest cell-mesoderm interface, for reviews, [13], [14], [49]. Our quail into chick transplantations combined with our molecular analysis using markers for both cell types were not consistent with a sharp interface between neural crest cells and cephalic paraxial mesoderm. We clearly observed the presence of neural crest cells (*AP2α*-positive or of quail origin after our transplantation studies) within the cephalic mesoderm expressing *MyoR* (Figure 3,4), highlighting a previously undescribed intermingling of both cell types and the absence of a clear frontier between neural crest cells and cephalic mesoderm. This tight association could reflect intimate interactions between neural crest cells and cephalic paraxial mesoderm at early stages of branchial development. Indeed cranial (versus trunk) mesoderm has permissive properties for allowing cranial neural crest cell migration [22], [43], [50]. Conversely, ablation of neural crest cells has been shown to affect cephalic paraxial mesoderm invasion of the branchial arches [27]. The surrounding environment has been shown to be determinant for the proper migration of both neural crest cells [34] and cephalic mesoderm [9] to fill the branchial arches. Early intermingling of neural crest cells and cephalic mesoderm is consistent with the idea that both cell populations respond to the same extrinsic cues for cell migration, at least at the level of the first branchial arch. However, the mechanisms and the molecular signals responsible for the coherent migration of cranial neural crest cells and cephalic mesoderm to the specific branchial arches remain to be elucidated [51].

As soon as the first branchial arch forms (at around the 21 somite-stage) there is a striking expansion of neural crest cell populations surrounding the myogenic core relative to neural crest cells within the core. This leads to a dramatic increase of neural crest cell density around the core within 24 hours (between HH13/E2 and HH20/E3). One hypothesis for this increase is that the surface ectoderm and pharyngeal endoderm provide proliferative signal(s) to induce proliferation of underlying neural crest cells. One obvious candidate for inducing neural crest cell proliferation is the Fgf8 signal located in arch ectoderm and endoderm, since a trophic and/or survival effect of Fgf8 on neural crest cells has been well documented using various genetic models and manipulations in chick embryos [52]–[54]. Shh is another candidate, since Shh promotes the development of multipotent neural crest cell progenitors [55]–[57]. Neural crest cells inside the myogenic core would be unresponsive to such proliferative signals, either by a passive mechanism such as being protected by the future myogenic cells or by an unknown inhibitory signal from the same cells. We hypothesise that the few neural crest cells observed in the myogenic core of the first branchial arch at HH21 (E3.5) are those observed at earlier stages. The neural crest cells within the early mesodermal core are likely to be the progenitors of muscle connective tissue cells and could play important roles in later branchiomeric muscle organisation. Furthermore, these neural crest cells may also be involved in myogenic differentiation, as they are present in the mesodermal core during the early step of myogenesis, before the myogenic program, assayed by *MyoD* expression, is activated. Indeed, neural crest cells within the core express the Wnt antagonist secreted factor, *Frzb-1* [58], which has been shown to promote the formation of vertebrate head muscle [26]. The presence of *Frzb-1* inside the myogenic core could provide a favourable environment for muscle differentiation.

The intermingling of neural crest cells and cephalic mesoderm in the future branchial arches is consistent with the congruence of skeletal and muscle elements in the jaw, since the neural crest cells and cephalic mesoderm at the same antero-posterior position share common destinations [8], [59]–[61]. The congruence also applies to branchiomeric innervation; since all these muscle precursors arise at the same axial levels as their corresponding motor axons [60]. Moreover, neural crest cells have been shown to be involved in the targeting of nerve exit points [62], [63]. Here again, the presence of neural crest cells mixed within cephalic mesoderm, which will later give rise to branchiomeric muscles, is consistent with the congruence of muscle and nerve components.

Muscle splitting occurs progressively from HH26 (E5), leading to the formation of individual branchiomeric muscles derived from the first arch [14], [49], [59]. The cellular and molecular mechanisms underlying the splitting process of arch muscles are not known. Although there is evidence that muscle connective tissue, tendons and vessels are involved in limb muscle segregation [38], [64], [65], no such evidence exists for the splitting of branchiomeric muscles. Once branchiomeric muscles are individualised, neural crest-derived cells surround the separated muscles. Although ablation of neural folds in chick model leads to severe muscle defects, suggesting an involvement of neural crest cells in muscle patterning [27], there is as yet no demonstration of a direct instructive role of cranial neural crest cells in controlling branchiomeric muscle segregation. Concomitant with muscle splitting, there is an increase of neural crest cell number inside muscles from HH27 (E5.5) as compared to earlier stages (Figure 5). These neural crest cells are not labelled by the tendon marker *Scleraxis* and may provide muscle connective tissue cells. It has previously been proposed that the neural crest cells providing muscle connective tissue elements penetrate the muscle masses from HH27/28 [14]. However, given that neural crest cells are present within the myogenic core from earlier stages (this paper), we do not favour cell infiltration but rather neural crest cell proliferation inside the muscle masses. The molecular mechanisms triggering this increase of neural crest cells inside the muscle masses from HH27 is not known. However, this increase of neural crest cells within the arch muscles is correlated with the appearance of tendon primordia expressing *Scleraxis* surrounding the muscle masses.

In summary, it has been shown independently that cranial mesoderm and neural crest cells have intrinsic properties along the antero-posterior axis, but their migration, patterning and differentiation depend on extrinsic cues (reviewed in [22] for cranial mesoderm and in [66] for neural crest cells). The extrinsic cues patterning the branchial arches are provided by the surface ectoderm and pharyngeal endoderm [56], [67]–[71]. Our results suggest that these common extrinsic cues may lead to intermingling of neural crest cells and cephalic mesoderm at early stages of branchial arch development.

### Tendon and muscle interactions in the branchial arches

We have shown that *Scleraxis* labels all the tendons associated with extra-ocular and branchiomeric muscles in chick and mouse embryos. In addition, the *Scleraxis-*positive cells associated with eye and branchiomeric muscles are of neural crest cell origin. Finally, using a genetic perturbation of branchiomeric muscle formation in mouse embryos, we have shown that *Scleraxis* expression is initiated independently of branchiomeric muscles, but is not maintained in the absence of branchiomeric muscles. This shows that one neural crest cell-derived tissue, tendon, initiates its development independently of muscles in branchial arches. In *Tbx1* mutant mice, branchiomeric myogenesis fails, based on the absence of myogenic regulatory factor activation and lack of subsequent muscle formation. *Capsulin* and *MyoR* expression is initiated in the first branchial arch at E9.5 in the absence of *Tbx1* activity, indicating the presence of a pre-myogenic mesodermal core [17], [32]. However, *MyoR* expression is down-regulated in the first branchial arch of *Tbx1^−/−^* embryos by E11 before the appearance of *Scleraxis* expression in tendon primordia (data not shown), indicating that *MyoR* and *Scleraxis* expression in tendon primordia are not detected at same time in *Tbx1^−/−^* mutant mice. This argues against an early signal from the pre-myogenic mesodermal cells triggering the establishment and patterning of *Scleraxis*-positive cells, indicating that tendon development initiates normally in *Tbx1* mutant embryos. In addition, we have also shown that, at later stages, *Scleraxis* expression is lost in the absence of branchiomeric muscles. This shows that tendons do not continue their development in the absence of branchiomeric muscles. The muscle-independence of tendon initiation and the later muscle requirement for further tendon development is similar to the situation in the limb, where *Scleraxis* expression is normally detected in tendon primordia in muscleless limbs in chick and mouse embryos, but is progressively lost in the absence of limb muscles [39], [72], [73]. It is important to point out that in the context of branchial development, it is the tendon precursor cells originating from the crest that migrate into the cephalic mesoderm containing the muscle precursors, while in the limb it is the muscle precursor cells that migrate into limb mesenchyme, which provides tendon cells. Despite this fundamental difference, tendon and muscle cell interactions are similar in both branchial arches and limbs. Interestingly, this type of muscle and tendon interaction also operates in *Drosophila*, where tendon precursor cells are specified in the absence of muscles, but muscle attachment is required for subsequent differentiation of *Drosophila* tendon cells [74], [75].

Muscle development has been studied in the absence of neural crest cells. The early steps of branchiomeric muscle formation are independent of the presence of the neural crest cells but, in the absence of neural crest cells, muscle formation is severely impaired [21], [27]. Combined with our studies of the influence of muscle on the formation of one neural crest derived tissue (tendon), this shows that both tendon and muscle precursors of the branchial arches initiate their development independently of each other, and that reciprocal interactions are necessary for further development of both cell types.

In conclusion, during branchial arch development, neural crest cells providing cartilage, connective tissues and tendons are intricately mixed with cephalic mesoderm providing muscle cells, both cell populations probably responding to the same extrinsic cues. Despite this intermingling between neural crest and mesodermal cells, the initial steps of the formation of one neural crest cell-derivative, tendons, and of muscles are independent of each other. However, late steps of tendon and muscle development require reciprocal interactions.

## References

[1] Cerny R, Meulemans D, Berger J, Wilsch-Brauninger M, Kurth T (2004). Combined intrinsic and extrinsic influences pattern cranial neural crest migration and pharyngeal arch morphogenesis in axolotl.. Dev Biol.

[2] Lee SH, Bedard O, Buchtova M, Fu K, Richman JM (2004). A new origin for the maxillary jaw.. Dev Biol.

[3] Le Lievre CS, Le Douarin NM (1975). Mesenchymal derivatives of the neural crest: analysis of chimaeric quail and chick embryos.. J Embryol Exp Morphol.

[4] Noden DM (1983a). The embryonic origins of avian cephalic and cervical muscles and associated connective tissues.. Am J Anat.

[5] Couly GF, Coltey PM, Le Douarin NM (1992). The developmental fate of the cephalic mesoderm in quail-chick chimeras.. Development.

[6] Le Douarin NM, Ziller C, Couly GF (1993). Patterning of neural crest derivatives in the avian embryo: in vivo and in vitro studies.. Dev Biol.

[7] Trainor PA, Tan SS, Tam PP (1994). Cranial paraxial mesoderm: regionalisation of cell fate and impact on craniofacial development in mouse embryos.. Development.

[8] Trainor PA, Tam PP (1995). Cranial paraxial mesoderm and neural crest cells of the mouse embryo: co-distribution in the craniofacial mesenchyme but distinct segregation in branchial arches.. Development.

[9] Hacker A, Guthrie S (1998). A distinct developmental programme for the cranial paraxial mesoderm in the chick embryo.. Development.

[10] Evans DJ, Noden DM (2006). Spatial relations between avian craniofacial neural crest and paraxial mesoderm cells.. Dev Dyn.

[11] Couly GF, Coltey PM, Le Douarin NM (1993). The triple origin of skull in higher vertebrates: a study in quail-chick chimeras.. Development.

[12] Kontges G, Lumsden A (1996). Rhombencephalic neural crest segmentation is preserved throughout craniofacial ontogeny.. Development.

[13] Noden DM, Trainor PA (2005). Relations and interactions between cranial mesoderm and neural crest populations.. J Anat.

[14] Noden DM, Francis-West P (2006). The differentiation and morphogenesis of craniofacial muscles.. Dev Dyn.

[15] Grifone R, Kelly RG (2007). Heartening news for head muscle development.. Trends Genet.

[16] Lu JR, Bassel-Duby R, Hawkins A, Chang P, Valdez R (2002). Control of facial muscle development by MyoR and capsulin.. Science.

[17] Kelly RG, Jerome-Majewska LA, Papaioannou VE (2004). The del22q11.2 candidate gene Tbx1 regulates branchiomeric myogenesis.. Hum Mol Genet.

[18] Dastjerdi A, Robson L, Walker R, Hadley J, Zhang Z (2007). Tbx1 regulation of myogenic differentiation in the limb and cranial mesoderm.. Dev Dyn.

[19] Dong F, Sun X, Liu W, Ai D, Klysik E (2006). Pitx2 promotes development of splanchnic mesoderm-derived branchiomeric muscle.. Development.

[20] Shih HP, Gross MK, Kioussi C (2007). Cranial muscle defects of Pitx2 mutants result from specification defects in the first branchial arch.. Proc Natl Acad Sci U S A.

[21] von Scheven G, Alvares LE, Mootoosamy RC, Dietrich S (2006a). Neural tube derived signals and Fgf8 act antagonistically to specify eye versus mandibular arch muscles.. Development.

[22] Bothe I, Ahmed MU, Winterbottom FL, von Scheven G, Dietrich S (2007). Extrinsic versus intrinsic cues in avian paraxial mesoderm patterning and differentiation.. Dev Dyn.

[23] Noden DM (1983b). The role of the neural crest in patterning of avian cranial skeletal, connective, and muscle tissues.. Dev Biol.

[24] Schilling TF, Kimmel CB (1997). Musculoskeletal patterning in the pharyngeal segments of the zebrafish embryo.. Development.

[25] Olsson L, Falck P, Lopez K, Cobb J, Hanken J (2001). Cranial neural crest cells contribute to connective tissue in cranial muscles in the anuran amphibian, Bombina orientalis.. Dev Biol.

[26] Tzahor E, Kempf H, Mootoosamy RC, Poon AC, Abzhanov A (2003). Antagonists of Wnt and BMP signaling promote the formation of vertebrate head muscle.. Genes Dev.

[27] Rinon A, Lazar S, Marshall H, Buchmann-Moller S, Neufeld A (2007). Cranial neural crest cells regulate head muscle patterning and differentiation during vertebrate embryogenesis.. Development.

[28] Schweitzer R, Chyung JH, Murtaugh LC, Brent AE, Rosen V (2001). Analysis of the tendon cell fate using Scleraxis, a specific marker for tendons and ligaments.. Development.

[29] Brent AE, Schweitzer R, Tabin CJ (2003). A somitic compartment of tendon progenitors.. Cell.

[30] Pryce BA, Brent AE, Murchison ND, Tabin CJ, Schweitzer R (2007). Generation of transgenic tendon reporters, ScxGFP and ScxAP, using regulatory elements of the scleraxis gene.. Dev Dyn.

[31] Murchison ND, Price BA, Conner DA, Keene DR, Olson EN (2007). Regulation of tendon differentiation by scleraxis distinguishes force-transmitting tendons from muscle-anchoring tendons.. Development.

[32] Grifone R, Jarry T, Dandonneau M, Grenier J, Duprez D (2008). Properties of branchiomeric and somite-derived muscle development in Tbx1 mutant embryos.. Dev Dyn.

[33] Hamburger V, Hamilton HL (1992). A series of normal stages in the development of the chick embryo. 1951.. Dev Dyn.

[34] Le Douarin NM, Kalcheim C (1999). The Neural Crest..

[35] Creuzet S, Couly G, Vincent C, Le Douarin NM (2002). Negative effect of Hox gene expression on the development of the neural crest-derived facial skeleton.. Development.

[36] Jerome LA, Papaioannou VE (2001). DiGeorge syndrome phenotype in mice mutant for the T-box gene, Tbx1.. Nat Genet.

[37] Hadchouel J, Tajbakhsh S, Primig M, Chang TH, Daubas P (2000). Modular long-range regulation of Myf5 reveals unexpected heterogeneity between skeletal muscles in the mouse embryo.. Development.

[38] Tozer S, Bonnin MA, Relaix F, Di Savino S, Garcia-Villalba P (2007). Involvement of vessels and PDGFB in muscle splitting during chick limb development.. Development.

[39] Bonnin MA, Laclef C, Blaise R, Eloy-Trinquet S, Relaix F (2005). Six1 is not involved in limb tendon development, but is expressed in limb connective tissue under Shh regulation.. Mech Dev.

[40] von Scheven G, Bothe I, Ahmed MU, Alvares LE, Dietrich S (2006b). Protein and genomic organisation of vertebrate MyoR and Capsulin genes and their expression during avian development.. Gene Expr Patterns.

[41] Boardman PE, Sanz-Ezquerro J, Overton IM, Burt DW, Bosch E (2002). A comprehensive collection of chicken cDNAs.. Curr Biol.

[42] Tucker GC, Aoyama H, Lipinski M, Tursz T, Thiery JP (1984). Identical reactivity of monoclonal antibodies HNK-1 and NC-1: conservation in vertebrates on cells derived from the neural primordium and on some leukocytes.. Cell Differ.

[43] Mootoosamy RC, Dietrich S (2002). Distinct regulatory cascades for head and trunk myogenesis.. Development.

[44] Schorle H, Meier P, Buchert M, Jaenisch R, Mitchell PJ (1996). Transcription factor AP-2 essential for cranial closure and craniofacial development.. Nature.

[45] Zhang J, Hagopian-Donaldson S, Serbedzija G, Elsemore J, Plehn-Dujowich D (1996). Neural tube, skeletal and body wall defects in mice lacking transcription factor AP-2.. Nature.

[46] Shen H, Wilke T, Ashique AM, Narvey M, Zerucha T (1997). Chicken transcription factor AP-2: cloning, expression and its role in outgrowth of facial prominences and limb buds.. Dev Biol.

[47] Nottoli T, Hagopian-Donaldson S, Zhang J, Perkins A, Williams T (1998). AP-2-null cells disrupt morphogenesis of the eye, face, and limbs in chimeric mice.. Proc Natl Acad Sci U S A.

[48] Mitchell PJ, Timmons PM, Hebert JM, Rigby PW, Tjian R (1991). Transcription factor AP-2 is expressed in neural crest cell lineages during mouse embryogenesis.. Genes Dev.

[49] Noden DM, Marcucio R, Borycki AG, Emerson CP (1999). Differentiation of avian craniofacial muscles: I. Patterns of early regulatory gene expression and myosin heavy chain synthesis.. Dev Dyn.

[50] Noden DM (1986). Patterning of avian craniofacial muscles.. Dev Biol.

[51] Kulesa PM, Teddy JM, Stark DA, Smith SE, McLennan R (2008). Neural crest invasion is a spatially-ordered progression into the head with higher cell proliferation at the migratory front as revealed by the photoactivatable protein, KikGR.. Dev Biol.

[52] Trumpp A, Depew MJ, Rubenstein JL, Bishop JM, Martin GR (1999). Cre-mediated gene inactivation demonstrates that FGF8 is required for cell survival and patterning of the first branchial arch.. Genes Dev.

[53] Abu-Issa R, Smyth G, Smoak I, Yamamura K, Meyers EN (2002). Fgf8 is required for pharyngeal arch and cardiovascular development in the mouse.. Development.

[54] Creuzet S, Schuler B, Couly G, Le Douarin NM (2004). Reciprocal relationships between Fgf8 and neural crest cells in facial and forebrain development.. Proc Natl Acad Sci U S A.

[55] Ahlgren SC, Bronner-Fraser M (1999). Inhibition of sonic hedgehog signaling in vivo results in craniofacial neural crest cell death.. Curr Biol.

[56] Brito JM, Teillet MA, Le Douarin NM (2006). An early role for sonic hedgehog from foregut endoderm in jaw development: ensuring neural crest cell survival.. Proc Natl Acad Sci U S A.

[57] Calloni GW, Glavieux-Pardanaud C, Le Douarin NM, Dupin E (2007). Sonic Hedgehog promotes the development of multipotent neural crest progenitors endowed with both mesenchymal and neural potentials.. Proc Natl Acad Sci U S A.

[58] Duprez D, Leyns L, Bonnin MA, Lapointe F, Etchevers H (1999). Expression of Frzb-1 during chick development.. Mech Dev.

[59] McClearn D, Noden DM (1988). Ontogeny of architectural complexity in embryonic quail visceral arch muscles.. Am J Anat.

[60] Lumsden A, Keynes R (1989). Segmental patterns of neuronal development in the chick hindbrain.. Nature.

[61] Lumsden A, Sprawson N, Graham A (1991). Segmental origin and migration of neural crest cells in the hindbrain region of the chick embryo.. Development.

[62] Niederlander C, Lumsden A (1996). Late emigrating neural crest cells migrate specifically to the exit points of cranial branchiomotor nerves.. Development.

[63] Guthrie S (2007). Patterning and axon guidance of cranial motor neurons.. Nat Rev Neurosci.

[64] Kardon G (1998). Muscle and tendon morphogenesis in the avian hind limb.. Development.

[65] Kardon G, Harfe BD, Tabin CJ (2003). A Tcf4-positive mesodermal population provides a prepattern for vertebrate limb muscle patterning.. Dev Cell.

[66] Le Douarin NM, Creuzet S, Couly G, Dupin E (2004). Neural crest cell plasticity and its limits.. Development.

[67] Hu D, Helms JA (1999). The role of sonic hedgehog in normal and abnormal craniofacial morphogenesis.. Development.

[68] Lee SH, Fu KK, Hui JN, Richman JM (2001). Noggin and retinoic acid transform the identity of avian facial prominences.. Nature.

[69] Haworth KE, Wilson JM, Grevellec A, Cobourne MT, Healy C (2007). Sonic hedgehog in the pharyngeal endoderm controls arch pattern via regulation of Fgf8 in head ectoderm.. Dev Biol.

[70] Brito JM, Teillet MA, Le Douarin NM (2008). Induction of mirror-image supernumerary jaws in chicken mandibular mesenchyme by Sonic Hedgehog-producing cells.. Development.

[71] Szabo-Rogers HL, Geetha-Loganathan P, Nimmagadda S, Fu KK, Richman JM (2008). FGF signals from the nasal pit are necessary for normal facial morphogenesis.. Dev Biol.

[72] Edom-Vovard F, Schuler B, Bonnin MA, Teillet MA, Duprez D (2002). Fgf4 positively regulates scleraxis and tenascin expression in chick limb tendons.. Dev Biol.

[73] Edom-Vovard F, Duprez D (2004). Signals regulating tendon formation during chick embryonic development.. Dev Dyn.

[74] Volk T (1999). Singling out Drosophila tendon cells: a dialogue between two distinct cell types.. Trends Genet.

[75] Volohonsky G, Edenfeld G, Klambt C, Volk T (2007). Muscle-dependent maturation of tendon cells is induced by post-transcriptional regulation of stripeA.. Development.

